# Scikit-Dimension: A Python Package for Intrinsic Dimension Estimation

**DOI:** 10.3390/e23101368

**Published:** 2021-10-19

**Authors:** Jonathan Bac, Evgeny M. Mirkes, Alexander N. Gorban, Ivan Tyukin, Andrei Zinovyev

**Affiliations:** 1 Institut Curie, PSL Research University, 75248 Paris, France; 2 INSERM, U900, 75248 Paris, France; 3 CBIO-Centre for Computational Biology, Mines ParisTech, PSL Research University, 75272 Paris, France; 4 Department of Mathematics, University of Leicester, Leicester LE1 7RH, UK; em322@leicester.ac.uk (E.M.M.); a.n.gorban@leicester.ac.uk (A.N.G.); i.tyukin@leicester.ac.uk (I.T.); 5 Laboratory of Advanced Methods for High-Dimensional Data Analysis, Lobachevsky University, 603105 Nizhniy Novgorod, Russia

**Keywords:** intrinsic dimension, effective dimension, Python package, method benchmarking

## Abstract

Dealing with uncertainty in applications of machine learning to real-life data critically depends on the knowledge of intrinsic dimensionality (ID). A number of methods have been suggested for the purpose of estimating ID, but no standard package to easily apply them one by one or all at once has been implemented in Python. This technical note introduces scikit-dimension, an open-source Python package for intrinsic dimension estimation. The scikit-dimension package provides a uniform implementation of most of the known ID estimators based on the scikit-learn application programming interface to evaluate the global and local intrinsic dimension, as well as generators of synthetic toy and benchmark datasets widespread in the literature. The package is developed with tools assessing the code quality, coverage, unit testing and continuous integration. We briefly describe the package and demonstrate its use in a large-scale (more than 500 datasets) benchmarking of methods for ID estimation for real-life and synthetic data.

## 1. Introduction

We present scikit-dimension, an open-source Python package for global and local intrinsic dimension (ID) estimation. The package has two main objectives: (i) foster research in ID estimation by providing code to benchmark algorithms and a platform to share algorithms; and (ii) democratize the use of ID estimation by providing user-friendly implementations of algorithms using the scikit-learn application programming interface (API) [1].

ID intuitively refers to the minimum number of parameters required to represent a dataset with satisfactory accuracy. The meaning of “accuracy” can be different among various approaches. ID can be more precisely defined to be *n* if the data lie closely to a *n*-dimensional manifold embedded in $R^{d}$ with little information loss, which corresponds to the so-called “manifold hypothesis” [2,3]. ID can be, however, defined without assuming the existence of a data manifold. In this case, data point cloud characteristics (e.g., linear separability or pattern of covariance) are compared to a model *n*-dimensional distribution (e.g., uniformly sampled *n*-sphere or *n*-dimensional isotropic Gaussian distribution), and the term “effective dimensionality” is sometimes used instead of “intrinsic dimensionality” as such *n* giving the most similar characteristics to the one measured in the studied point cloud [4,5]. In scikit-dimension, these two notions are not distinguished.

The knowledge of ID is important to determine the choice of machine learning algorithm, anticipate the uncertainty of its predictions, and estimate the number of sufficiently distinct clusters of variables [6,7]. The well-known *curse of dimensionality*, which states that many problems become exponentially difficult in high dimensions, does not depend on the number of features, but on the dataset’s ID [8]. More precisely, the effects of the dimensionality curse are expected to be manifested when ID≫ln(M), where *M* is the number of data points [9,10].

Current ID estimators have diverse operating principles (we refer the reader to [11] for an overview). Each ID estimator is developed based on a selected feature (such as the number of data points in a sphere of fixed radius, linear separability or expected normalized distance to the closest neighbor), which scales with *n*: therefore, various ID estimation methods provide different ID values. Each dataset can be characterized by a unique *dimensionality profile* of ID estimations, according to different existing methods, which can serve as an important signature for choosing the most appropriate data analysis method.

Dimensionality estimators that provide a single ID value for the whole dataset belong to the category of global estimators. However, datasets can have complex organizations and contain regions with varying dimensionality [9]. In such a case, they can be explored using local estimators, which estimate ID in local neighborhoods around each point. The neighborhoods are typically defined by considering the *k* closest neighbors. Such approaches also allow repurposing global estimators as local estimators.

The idea behind local ID estimation is to operate at a scale where the data manifold can be approximated by its tangent space [12]. In practice, ID is sensitive to scale, and choosing the neighborhood size is a trade-off between opposite requirements [11,13]: ideally, the neighborhood should be big relative to the scale of the noise, and contain enough points. At the same time, it should be small enough to be well approximated by a flat and uniform tangent space.

We perform benchmarking of 19 ID estimators on a large collection of real-life and synthetic datasets. Previously, estimators were benchmarked based mainly on artificial datasets representing uniformly sampled manifolds with known ID [4,11,14], comparing them for the ability to estimate the ID value correctly. Several ID estimators were used on real-life datasets to evaluate the degree of dimensionality curse in a study of various metrics in data space [15]. Here, we benchmark ID estimation methods, focusing on their applicability to a wide range of datasets of different origin, configuration and size. We also look at how different ID estimations are correlated, and show how scikit-dimension can be used to derive a consensus measure of data dimensionality by averaging multiple individual measures. The latter can be a robust measure of data dimensionality in various applications.

Scikit-dimension was applied in several recent studies for estimating the intrinsic dimensionality of real-life datasets [16,17].

## 2. Materials and Methods

### 2.1. Software Features

Scikit-dimension is an open-source software available at https://github.com/j-bac/scikit-dimension (accessed on 18 October 2021).

Scikit-dimension consists of two modules. The *id* module provides ID estimators, and the *datasets* module provides synthetic benchmark datasets.

#### 2.1.1. *id* Module

The *id* module contains estimators based on the following:

- Correlation (fractal) dimension (id.CorrInt) [18].
- Manifold-adaptive fractal dimension (id.MADA) [19].
- Method of moments (id.MOM) [20].
- Principal component analysis (id.lPCA) [3,21,22,23].
- Maximum likelihood (id.MLE) [24,25,26].
- Minimum spanning trees (id.KNN) [27].
- Estimators based on concentration of measure (id.MiND_ML, id.DANCo, id.ESS, id.TwoNN, id.FisherS, id.TLE) [4,28,29,30,31,32,33].

The description of the method principles is provided together with the package documentation at https://scikit-dimension.readthedocs.io/ (accessed on 18 October 2021) and in reviews [5,9,14].

#### 2.1.2. *Datasets* Module

**Figure 1:**
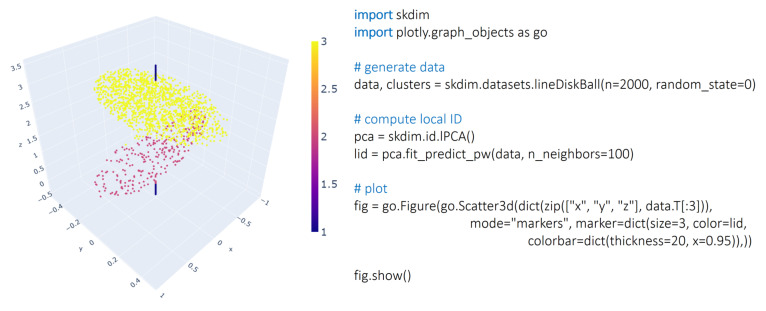
Example usage: generating the Line–Disk–Ball dataset [10]), which has clusters of varying local ID, and coloring points by estimates of local ID obtained by id.lPCA.

The *datasets* module allows user to test estimators on synthetic datasets; Figure 1. It can generate several low-dimensional toy datasets to play with different estimators as well as a set of synthetic manifolds commonly used to benchmark ID estimators, introduced by [14] and further extended in [11,28].

### 2.2. Development

Scikit-dimension is built according to the scikit-learn API [1] with support for Linux, MacOS, Windows and Python >= 3.6. The code style and API design are based on the guidelines of scikit-learn, with the NumPy [34] documentation format, and continuous integration on all three platforms. The online documentation is built using Sphinx and hosted with ReadTheDocs.

### 2.3. Dependencies

Scikit-dimension depends on a limited number of external dependencies on the user side for ease of installation and maintenance:

- Matplotlib [35]
- Pandas [36].
- Scikit-learn [1].
- Numba [37].
- SciPy [38]
- NumPy [34].

### 2.4. Related Software

Related open-source software for ID estimation have previously been developed in different languages such as R, MATLAB or C++ and contributed to the development of scikit-dimension.

In particular, refs. [10,39,40,41] provided extensive collections of ID estimators and datasets for R users, with [40] additionally focusing on dimension reduction algorithms. Similar resources can be found for MATLAB users [42,43,44,45]. Benchmarking many of the methods for ID estimation included in this package was performed in [15]. Finally, there exist several packages implementing standalone algorithms; in particular for Python, we refer the reader to complementary implementations of the GeoMLE, full correlation dimension, and GraphDistancesID algorithms [46,47,48,49].

To our knowledge, scikit-dimension is the first Python implementation of an extensive collection of ID methods. Compared to similar efforts in other languages, the package puts emphasis on estimators, quantifying various properties of high-dimensional data geometry, such as the concentration of measure. It is the only package to include ID estimation based on linear separability of data, using Fisher discriminants [4,32,50,51].

## 3. Results

### 3.1. Benchmarking Scikit-Dimension on a Large Collection of Datasets

In order to demonstrate the applicability of scikit-dimension to a wide range of real-life datasets of various configurations and sizes, we performed a large-scale benchmarking of scikit-dimension, using the collection of datasets from the OpenML repository [52]. We selected those datasets having at least 1000 observations and 10 features, without missing values. We excluded those datasets which were difficult to fetch, either because of their size or an error in the OpenML API. After filtering out repetitive entries, 499 datasets were collected. Their number of observations varied from 1002 to 9,199,930, and their number of features varied from 10 to 13,196. We focused only on numerical variables, and we subsampled the number of rows in the matrix to a maximum of 100,000. All dataset features were scaled to unit interval using Min/Max scaling. In addition, we filtered out approximate non-unique columns and rows in the data matrices since some of the ID methods could be affected by the presence of identical (or approximately identical) rows or columns.

We added to the collection 18 datasets, containing single-cell transcriptomic measurements, from the CytoTRACE study [53] and 4 largest datasets from The Cancer Genome Atlas (TCGA), containing bulk transcriptomic measurements. Therefore, our final collection contained 521 datasets altogether.

#### 3.1.1. Scikit-Dimension ID Estimator Method Features

We systematically applied 19 ID estimation methods from scikit-dimension, with default parameter values, including 7 methods based on application of principal component analysis (“linear” or PCA-based ID methods), and 12 based on application of various other principles, including correlation dimension and concentration of measure-based methods (“nonlinear” ID methods).

For KNN and MADA methods, we had to further subsample the data matrix to a maximum of 20,000 rows; otherwise they were too greedy in terms of memory consumption. Moreover, DANCo and ESS methods appeared to be too slow, especially in the case of a large number of variables: therefore, we made ID estimations in these cases on small fragments of data matrices. Thus, for DANCo, the maximum matrix size was set to 10,000 × 100, and for ESS to 2000 × 20. The number of features was reduced for these methods when needed, by using PCA-derived coordinates, and the number of observations were reduced by random subsampling.

**Figure 2:**
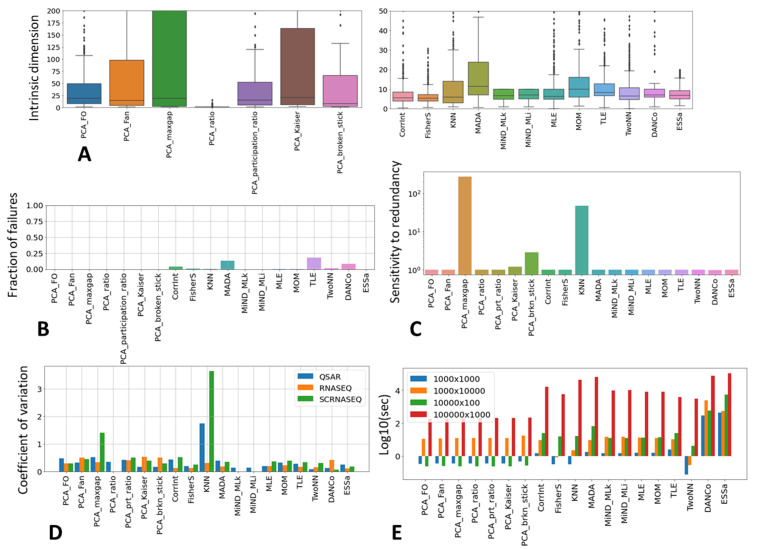
Illustrating different ID method general characteristics: (**A**) range of estimated ID values; (**B**) ability to produce interpretable (positive finite value) result; (**C**) sensitivity to feature redundancy (after duplicating matrix columns); (**D**) uniform ID estimation across datasets of similar nature; (**E**) computational time needed to compute ID for matrices of four characteristic sizes.

In Table 1, we provide the summary of characteristics of the tested methods. In more detail, the following method features were evaluated (see Figure 2).

Firstly, we simply looked at the ranges of ID values produced by the methods across all the datasets. These ranges varied significantly between the methods, especially for the linear ones (Figure 2A).

Secondly, we tested the methods with respect to their ability to successfully compute the ID as a positive finite value. It appeared that certain methods (such as MADA and TLE), in a certain number of cases produced a significant fraction of uninterpretable estimates (such as “nan” or negative value); Figure 2B. We assume that in most of such cases, the problem with ID estimation is caused by the method implementation, not anticipating certain relatively rare data point configurations, rather than the methodology itself, and that a reasonable ID estimate always exists. Therefore, in the case of an uninterpretable value due to method implementation, for further analysis, we considered it possible to impute the ID value from the results of application of other methods; see below.

Thirdly, for a small number of datasets, we performed a test of their sensitivity to the presence of strongly redundant features. For this purpose, we duplicated all features in a matrix and recomputed the ID. The resulting sensitivity is the ratio between the ID computed for the larger matrix and the ID computed for the initial matrix, having no duplicated columns. It appears that despite most of the methods being robust with respect to such a matrix duplication, some (such as PCA-based broken stick or the famous Kaiser methods popular in various fields, such as biology [54,55]), tend to be very sensitive (Figure 2C), which is compliant with some previous reports [15].

Some of the datasets in our collection could be combined in homogeneous groups according to their origin, such as the data coming from quantitative structure–activity relationship (QSAR)–based quantification of a set of chemicals. The size of the QSAR fingerprint for the molecules is the same in all such datasets (1024 features): therefore, we can assume that the estimate of ID will not vary too much across the datasets from the same group. We computed the coefficient of a variation of ID estimates across three such dataset groups, which revealed that certain methods tend to provide less stable estimations than the others; Figure 2D.

Finally, we recorded the computational time needed for each method. We found that the computational time could be estimated with good precision ($R^{2}>0.93$ for all ID estimators), using the multiplicative model: $Time=c\times N_{obj}^{\alpha }\times N_{var}^{\beta }$, where $N_{obj}$ and $N_{var}$ are the number of objects and features in a dataset, correspondingly. Using this model fit for each method, we estimated the time needed to estimate ID for data matrices of four characteristic sizes; Figure 2E.

#### 3.1.2. Metanalysis of Scikit-Dimension ID Estimates

After application of scikit-dimension, each dataset was characterized by a vector of 19 measurements of intrinsic dimensionality. The resulting matrix of ID values contained 2.5% missing values, which were imputed, using the standard IterativeImputer from the sklearn Python package.

**Figure 3:**
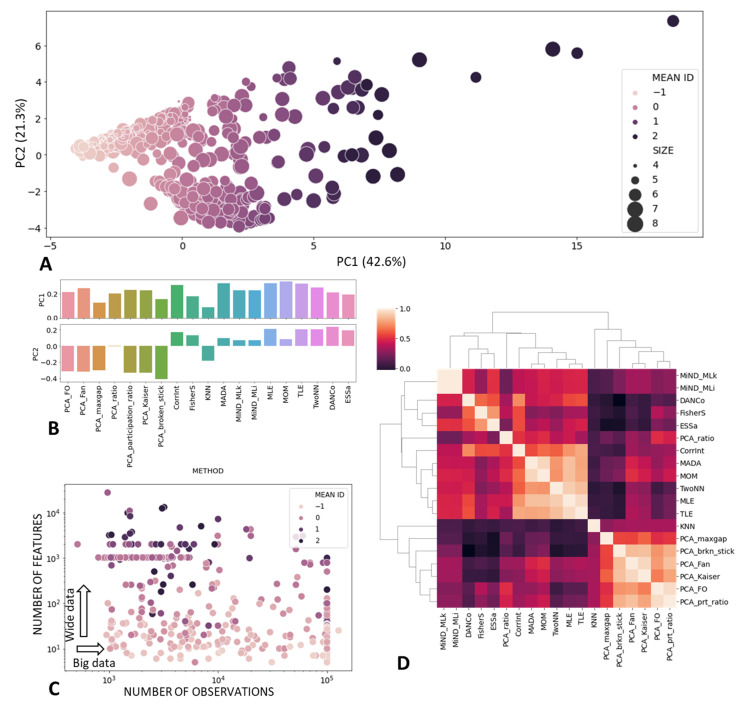
Characterizing OpenML dataset collection in terms of ID estimates. (**A**) PCA visualizations of datasets characterized by vectors of 19 ID measures. Size of the point corresponds to the logarithm of the number of matrix entries ($N_{obj}\times N_{var}$). The color corresponds to the mean ID estimate taken as the mean of all ID measure z-scores. (**B**) Loadings of various methods into the first and the second principal component from (**A**). (**C**) Visualization of the mean ID score as a function of data matrix shape. The color is the same as in (**A**). (**D**) Correlation matrix between different ID estimates computed over all analyzed datasets.

Using the imputed matrix and scaling it to z-scores, we performed principal component analysis (Figure 3A,B). The first principal component explained 42.6% percent of the total variance in ID estimations, with all of the methods having positive and comparable loadings to the first principal component. This justifies the computation of the “consensus” intrinsic dimension measure, which we define here as the mean value of individual ID estimate z-scores. Therefore, the mean ID can take negative or positive values, roughly dividing the datasets into “lower-dimensional” and “higher-dimensional” (Figure 3A,C). The consensus ID estimate weakly negatively correlated with the number of observations (Pearson ρ=−0.25, *p*-value = $10^{-9}$) and positively correlated with the number of features in the dataset (r = 0.44, *p*-value = $10^{-25}$). Nevertheless, even for the datasets with similar matrix shapes, the mean ID estimate could be quite different (Figure 3C).

The second principal component explained 21.3% of the total variance in ID estimates. The loadings of this component roughly differentiated between PCA-based ID estimates and non-linear ID estimation methods, with one exception in the case of the KNN method.

We computed the correlation matrix between the results of application of different ID methods (Figure 3D), which also distinguished two large groups of PCA-based and “non-linear” methods. Furthermore, non-linear methods were split into the group of methods, producing results similar to the correlation (fractal) dimension (CorrInt, MADA, MOM, TwoNN, MLE, TLE) and methods based on the concentration of measure phenomena (FisherS, ESS, DANCo, MiND_ML).

**Figure 4:**
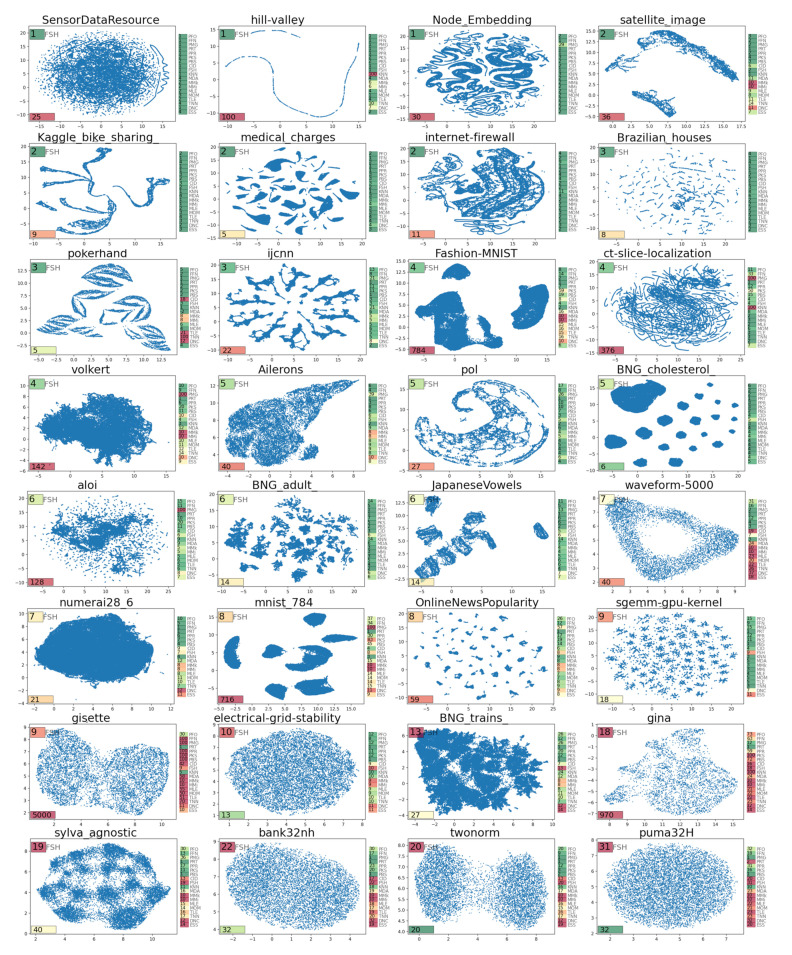
A gallery of UMAP plots computed for a selection of datasets from OpenML collection, with indication of ID estimates, ranked by the ID value estimated using Fisher separability-based method (indicated in the left top corner). The ambient dimension of the data (number of features $N_{var}$) is indicated in the bottom left corner, and the color reflects the $ID/N_{var}$ ratio, from red (close to 0.0 value) to green (close to 1.0). On the right from the UMAP plot, all 19 ID measures are indicated, with color mapped to the value range, from green (small dimension) to red (high dimension).

In order to illustrate the relation between the dataset geometry and the intrinsic dimension, we produced a gallery of uniform manifold approximation and projection (UMAP) dataset visualizations, with an indication of the ambient dataset dimension (number of features) and the estimated ID, using all methods; Figure 4. One of the conclusions that can be made from this analysis is that the UMAP visualization is not insightful for truly high-dimensional datasets (starting from ID = 10, estimated by the FisherS method). In addition, some datasets, having large ambient dimensions, were characterized with a low ID by most of the methods (e.g., ‘hill-valley’ dataset).

## 4. Conclusions

scikit-dimension is to our knowledge the first package implemented in Python, containing implementations of the most-used estimators of ID.

Benchmarking scikit-dimension on a large collection of real-life and synthetic datasets revealed that different estimators of ID possess internal consistency and that the ensemble of ID estimators allows us to achieve more robust classification of datasets into low- or high-dimensionality.

The estimation of intrinsic dimensionality of a dataset is essential in various applications of machine learning to real-life data. We can mention here several typical use cases, where the scikit-dimension package can be used, but this description is by no means comprehensive.

Firstly, learning low-dimensional data geometry (e.g., learning data manifolds or more complex geometries, such as principal graphs [60,61]) frequently requires preliminary data dimensionality reduction for which one has to estimate the ‘true’ global and local data dimensionality. For example, in the analysis of single-cell data in biology, the inference of so-called cellular trajectories can give different results when more or less principal data dimensions are kept. In higher dimensions, more cell fate decisions can be distinguished, but their inference becomes less robust [62,63]. Some advanced methods of unsupervised learning, such as quantifying the data manifold curvature, require knowledge of data ID [64]. In mathematical modeling of biological and other complex systems, it is frequently important to estimate the effective dimensionality of the dynamical process, from the data or from simulations, in order to inform model reduction [17,65,66]. In medical applications and in the analysis of clinical data, knowledge of consensus data dimensionality was shown to be important to distinguish signal from noise and predict patient trajectories [16].

Secondly, high-dimensional data geometry is a rapidly evolving field in machine learning [67,68,69]. To know whether the recent theoretical results can be used in practice, one has to estimate the ID of a concrete dataset. More generally, it is important to know if an application of a machine learning method to a dataset will face various types of difficulties, known as the curse of dimensionality. For example, it was shown that, under appropriate assumptions, robustness of general multi-class classifiers to adversarial examples can be achieved only if the intrinsic dimensionality of the AI’s decision variables is sufficiently small [70]. Knowledge of ID can be important to decide if one can benefit from the blessing of dimensionality in the problem of correcting the AI’s errors when deploying large, pre-trained legacy neural network models [32,71]. Estimating data dimensionality can suggest the application of specific data pre-processing methods, such as hubness reduction of point neighborhood graphs, in the tasks of clustering or non-linear dimensionality reduction [72]. In a recent study, estimating dataset ID was used to show that some old ideas on fighting the curse of dimensionality by modifying global data metrics are not efficient in practice [15]. In this respect, explicit control of the ID of AI models’ latent spaces appears to be crucial for developing robust and reliable AI. Our work adds to the spectrum of tools to achieve this aim.

Thirdly, local ID can be used to partition a data point cloud in a way that is complementary to standard clustering [73]. In 3D, this approach can be used for object detection (see Figure 1), but it can be generalized for higher-dimensional data point clouds. Interestingly, local ID can be related to various object characteristics in various domains: folded versus unfolded configurations in a protein molecular dynamics trajectory, active versus non-active regions in brain imaging data, and firms with different financial risk in company balance sheets [74].

Future releases of scikit-dimension will continuously seek to incorporate new estimators and benchmark datasets introduced in the literature, or new features, such as alternative nearest neighbor search for local ID estimates. The package will also include new ID estimators, which can be derived using the most recent achievements in understanding the properties of high-dimensional data geometry [71,75].

## Figures and Tables

**Table 1:** Summary table of ID methods characteristics. The qualitative score changes from “−−−” (worst) to “+++” (best).

Method Name	Short Name(s)	Ref(s)	Valid Result	Insensitivity to Redundancy	Uniform ID Estimate in Similar Datasets	Performance with Many Observations	Performance with Many Features
PCA Fukunaga-Olsen	PCA FO, PFO	[15,22]	+++	+++	+++	+++	+++
PCA Fan	PFN	[23]	+++	+++	+++	+++	+++
PCA maxgap	PMG	[56]	+++	−−−	+	+++	+++
PCA ratio	PRT	[57]	+++	+++	+	+++	+++
PCA participation ratio	PPR	[57]	+++	+++	++	+++	+++
PCA Kaiser	PKS	[54,58]	+++	−	+++	+++	+++
PCA broken stick	PBS	[55,59]	+++	−−	+++	+++	+++
Correlation (fractal) dimensionality	CorrInt, CID	[18]	+	+++	++	+	+
Fisher separability	FisherS, FSH	[4,32]	++	+++	+++	++	+++
K-nearest neighbours	KNN	[27]	++	−−	−−	−	++
Manifold-adaptive fractal dimension	MADA, MDA	[19]	−	+++	+++	−	+
Minimum neighbor distance—ML	MIND_ML,MMk, MMi	[28]	+++	+++	++	++	+
Maximum likelihood	MLE	[25]	++	+++	++	++	+
Methods of moments	MOM	[20]	+++	+++	+++	++	+
Estimation within tight localities	TLE	[33]	−−	+++	+++	++	+
Minimal neighborhood information	TwoNN,TNN	[31]	++	+++	+++	++	+++
Angle and norm concentration	DANCo,DNC	[29]	+	+++	+++	−−−	−−−
Expected simplex skewness	ESS	[56]	+++	+++	+++	−−−	−−−

## Data Availability

The datasets used in this study were retrieved from public sources, namely the OpenML repository, CytoTRACE website https://cytotrace.stanford.edu/ (section “Downloads”, accessed on 18 October 2021), from the Data Portal of National Cancer Institute https://portal.gdc.cancer.gov/ (accessed on 18 October 2021).
